# Metacarpophalangeal Joint Reconstruction of a Complex Hand Injury with a Vascularized Lateral Femoral Condyle Flap Using an Individualized 3D Printed Model—A Case Report

**DOI:** 10.3390/jpm13111570

**Published:** 2023-11-02

**Authors:** Michael Kohlhauser, Anna Vasilyeva, Lars-Peter Kamolz, Heinz K. Bürger, Michael Schintler

**Affiliations:** 1 Division of Plastic, Aesthetic and Reconstructive Surgery, Department of Surgery, Medical University of Graz, 8036 Graz, Austria; 2 COREMED—Cooperative Centre for Regenerative Medicine, Joanneum Research Forschungsgesellschaft mbH, 8010 Graz, Austria; 3 Division of Hand Surgery, Private Hospital Maria Hilf, 9020 Klagenfurt am Wörthersee, Austria

**Keywords:** hand surgery, microsurgery, reconstructive surgery, joint reconstruction, 3D printing, vascularized femoral condyle flap

## Abstract

This case report describes the surgical management of a patient with a complex hand trauma. This injury included tendon, vascular, and nerve injuries, a partial amputation of the index finger, fractures of the third proximal phalanx, and destruction of the metacarpophalangeal joint of the fifth finger. Firstly, the acute treatment of a complex hand injury is described. Secondly, the planning and execution of a joint reconstruction using a vascularized lateral femoral condylar flap, assisted by an individual 3D model, is illustrated. Precise reconstruction of the affected structures resulted in good revascularization as well as an anatomical bone consolidation. Intensive physical therapy, including autonomous proprioceptive range-of-motion exercises by the patient, resulted in significant functional improvement of the hand in daily life. Overall, we report on the successful reconstruction of a metacarpophalangeal joint by using a vascularized flap from the lateral femoral condyle. Furthermore, this case report highlights the efficacy of integrating individualized 3D printing technology to plan complex reconstructions, opening up promising opportunities for personalized and optimized interventions.

## 1. Introduction

The adequate treatment of severe hand injuries demands complex reconstruction, which is a frequent challenge. Due to the pronounced injury pattern with a large substance defect and possibly irreparable damage, primary reconstruction is often not viable and further interventions are indispensable for medical rehabilitation. However, often the regaining of full function is not achievable. According to a large cohort study, functional impairment occurs in 58.5% of all patients in the long-term follow-up control [1]. Thus, severe hand injuries can lead to significant repercussions, such as extended sick leave, enduring disabilities, and continued employment [2].

A recent advancement in reconstructive surgery is the development of individualized three-dimensional (3D) models for preoperative planning and simulation of surgical procedures. These printed models can provide a tangible representation of a complex deformity and help surgeons and patients better understand individual anatomical conditions by providing a visual and tactile aid. The usage of these techniques is expected to improve the surgical outcomes [3,4].

We present a case on the initial management and secondary reconstruction of a complex hand injury, which also includes the destruction of the fifth metacarpophalangeal (MCP) joint. In our case we report on the successful reconstruction of the fifth metacarpal bone with recovery of the functionality of the metacarpophalangeal joint using a vascularized flap from the lateral femoral condyle. For this reconstruction, a patient-personalized 3D-model was created to optimize pre- and intraoperative planning.

## 2. Case Report

**Figure 1:**
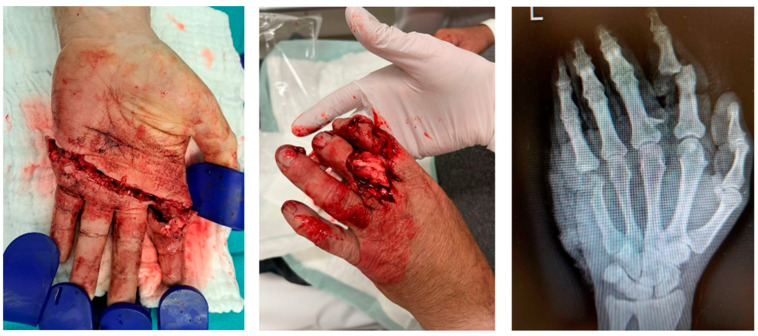
The initial findings in the emergency room.

A 57-year-old patient sustained a complex left hand injury while working with a circular saw. The injury included a partial amputation of the index finger, a fracture at the proximal phalanx of the third finger, and a comminuted fracture at the head of the fifth metacarpal bone. In addition, the superficial and deep flexor tendons from the second to the fifth finger were cleaved. Destruction of the third to the seventh and tenth proper palmar digital arteries by the saw-blade was also observed. Furthermore, the third to the eighth, and the tenth proper palmar digital nerves were severed. In Figure 1, both an image and an X-ray depicting the initial injury are discernible.

**Figure 2:**
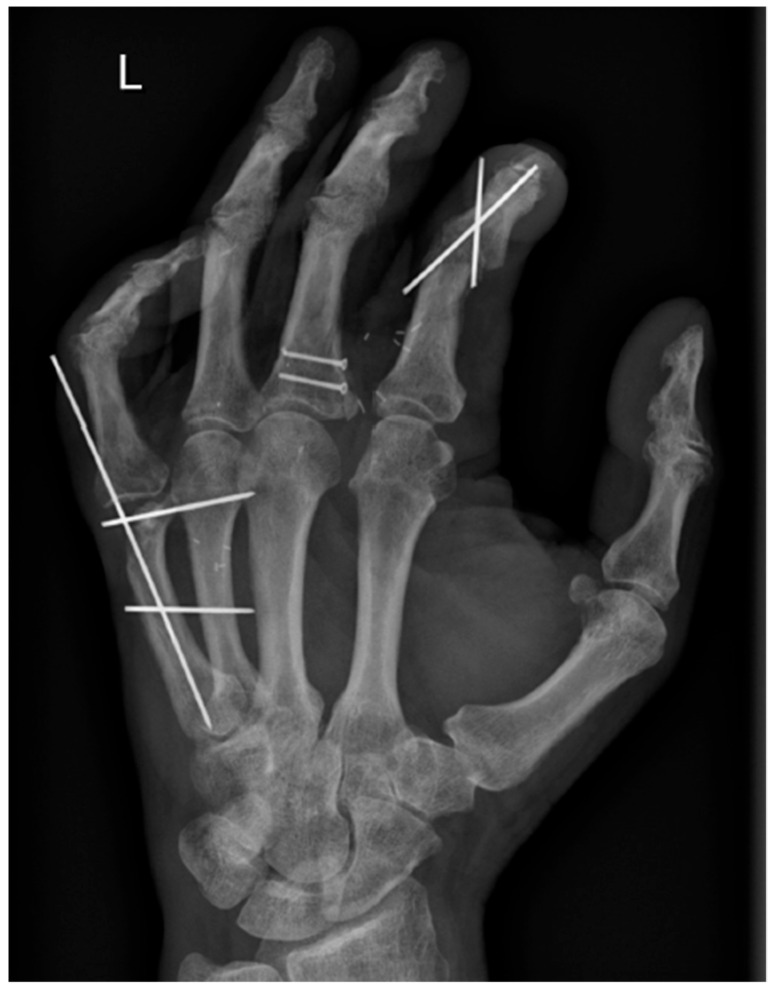
Postoperative X-ray image of the injured hand.

Initially, a screw osteosynthesis was performed on the proximal phalanx of the third finger. Due to the extent of injury to the fifth MCP joint, an intraoperative decision was made to secondarily reconstruct and temporarily stabilize it by the application of a three-pin osteosynthesis. Subsequently, the ulnar-collateral ligament and the joint capsule were sutured. A two-pin arthrodesis was then performed at the level of the proximal interphalangeal (PIP) joint on the left index. A postoperative X-ray image of the affected hand can be seen in Figure 2.

**Figure 3:**
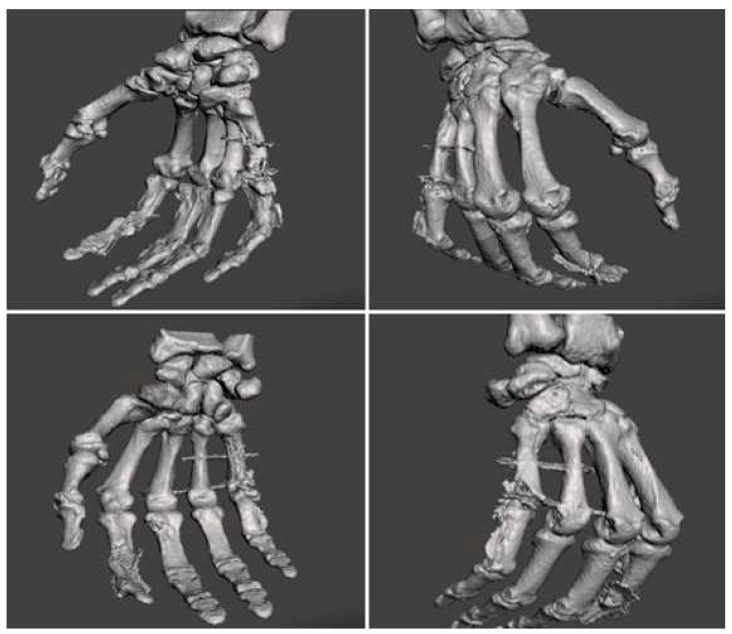
A 3D conversion of the CT images, created for preoperative planning.

After the release of a carpal tunnel, the first annular (A1) pulleys of the second to fifth fingers were cut to suture the deep flexor tendon of the affected fingers. As a next step, revascularization of the second finger was carried out using a micro-anastomosis of the third proper digital artery, followed by the neurorrhaphy of the third and fourth proper palmar digital nerves. Subsequently, the third finger underwent revascularization through a micro-anastomosis of the fifth proper digital artery, followed by neurorrhaphy of the fifth to the eighth and subsequently tenth proper digital nerves. Both the second and third fingers exhibit adequate revascularization with a physiological capillary refill time of approximately two seconds. Due to the extensive osseous defect in the fifth metacarpal, a two-stage reconstructive procedure was planned, slated for performance subsequent to the healing of the soft tissues. One week postoperatively, the patient was discharged with no complications. Two weeks after the trauma, physiotherapy could be initiated, taking the arthrodesis into account. One and a half months postoperatively, the patient demonstrates significant progress not only in wound healing but also in the mobility of the rehabilitated fingers. Considering the satisfactory progress, the reconstruction of the fifth metacarpal bone was initiated. Therefore, computed tomography (CT) imaging with conversion of a 3D model was performed to plan the reconstruction of the fifth MCP joint. The virtual 3D model can be seen in Figure 3.

**Figure 4:**
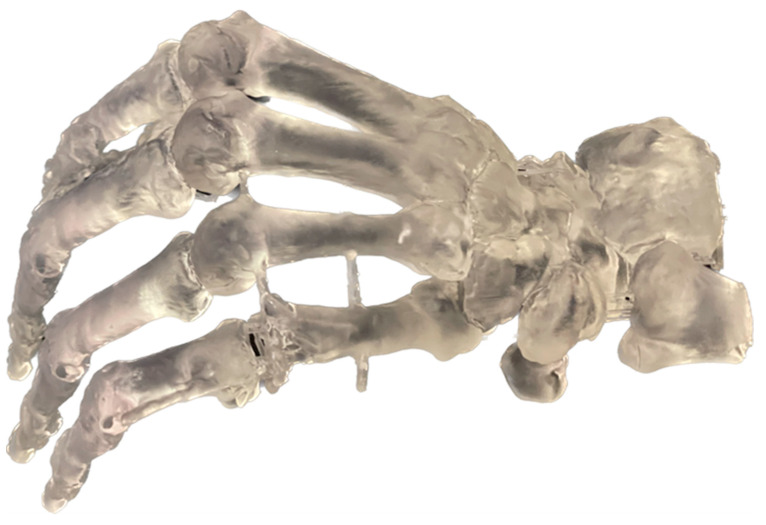
Image of the patient-specific replica, produced using a 3D printer for preoperative planning.

**Figure 5:**
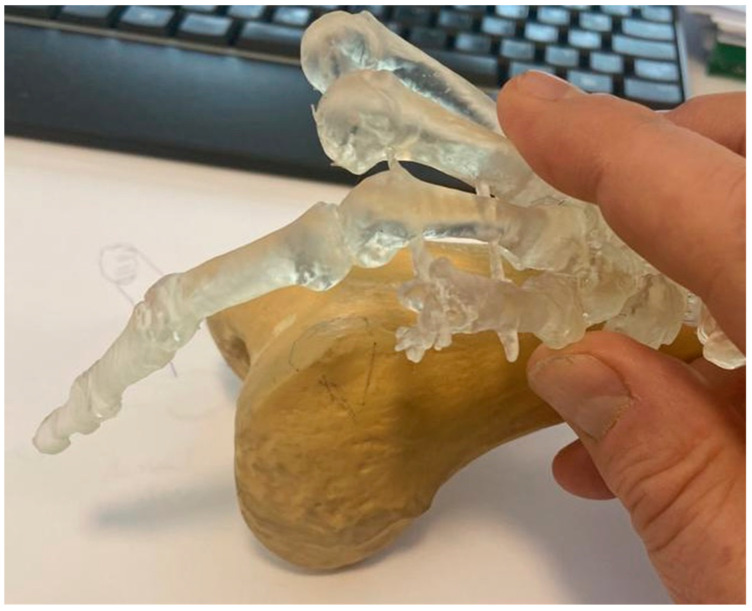
An image of the critical stage in preoperative planning taking into account the exact size and curvature of the fifth metacarpal bone head has occurred by using the individual replica from the 3D-printer.

With this 3D conversion of the CT imaging, an exact patient-specific replica of the defect could be produced from the 3D printer. Preoperative planning and simulation were then enabled by analysis of the individual anatomical conditions of the hand deformity. Initially, the reconstruction was planned with a vascularized flap from the medial femoral condyle; however, during the preoperative planning, the curvature of the lateral femur condyle proved to be a more appropriate substitution for the head of the fifth metacarpal bone. Consequently, a decision was taken to perform the reconstruction by using a vascularized lateral femoral condyle flap. Photographs of the individual replica and of the critical stage in preoperative planning are included in Figure 4 and Figure 5, respectively.

**Figure 6:**
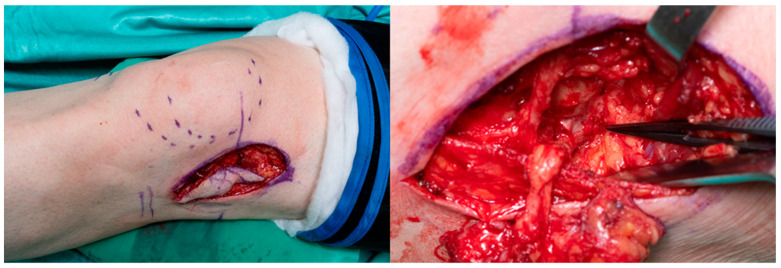
Intraoperative graft-take according to the exact size and curvature of the fifth metacarpal bone head by using the individual 3D replica. The superior lateral genicular artery serves as the flap pedicle.

After the removal of the pins and the arthrolysis, as well as the preparation of the connecting vessels for the reconstruction of the head of the fifth metacarpal bone, a vascularized femoral condyle flap was harvested from the non-weight bearing zone. This procedure was performed in accordance with the preoperative planning, taking into account the exact size and curvature of the fifth metacarpal bone head using the individual 3D replica. The flap pedicle consists of the superior lateral genicular artery with accompanying veins and its distal transverse branch, including a skin perforator, for defect coverage. An image of the intraoperative adaption is attached in Figure 6.

**Figure 7:**
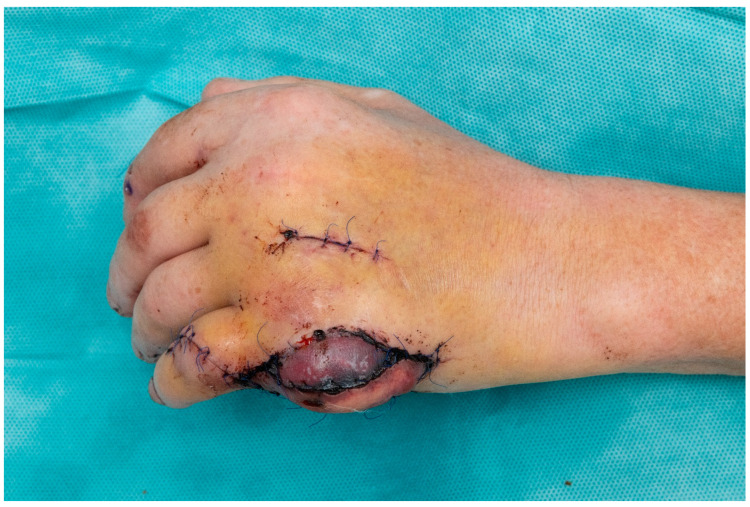
Postoperative image of the affected hand prior to the Epigard being replaced by a split-thickness skin graft.

The vascularized flap was fixed to the metacarpal bone by screw osteosynthesis. For the micro-anastomosis, the ninth digital propria artery served as the connecting artery, which was mobilized distally in the direction of the flap after dissection. A dorsal subcutaneous vein served as the venous anastomosis. A temporary wound closure was performed to avoid pressure on the micro anastomoses and flap vessels using Epigard, which was replaced with a split-thickness skin graft one week later. A postoperative image of the hand was attached in Figure 7.

**Figure 8:**
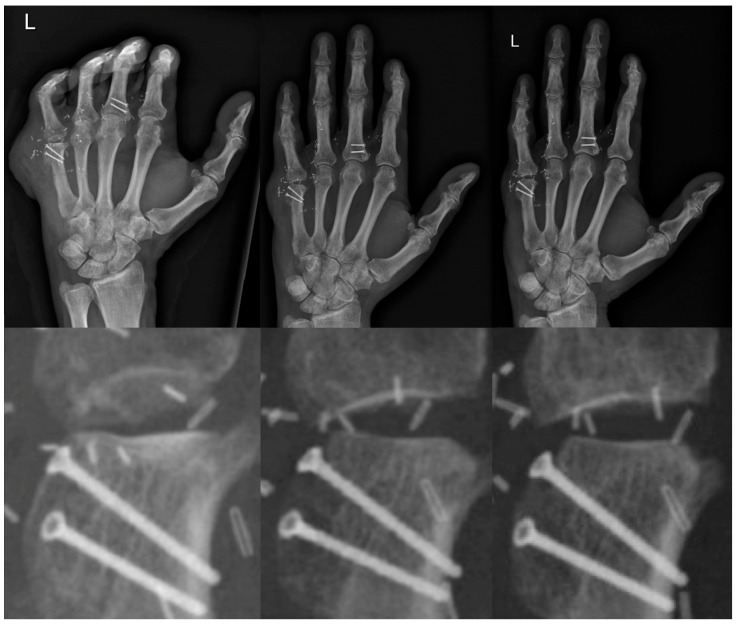
Overview and close-up of osseous consolidation progression, from the left to the right one day, two months and seven months postoperative.

Passive physical therapy commenced as early as 1 week and active 6-week after transplantation, with a weight-bearing ban imposed for 12 weeks postoperatively. During out-patient follow-ups, a physiological consolidation of the flap was observed in the X-ray imaging. The progression of the osseous consolidation has been depicted in Figure 8. Furthermore, significant functional improvement was evident following intensive physiotherapy, including the autonomous execution of proprioceptive exercises. Six months post-transplantation, despite minor limitations in the flexion and extension of the index and little finger, the affected hand demonstrates functional utility in daily activities, with subsequent evaluations indicating further improvement. The patient has successfully reintegrated into his professional environment and is able to perform his duties as a blacksmith with no limitations.

**Figure 9:**
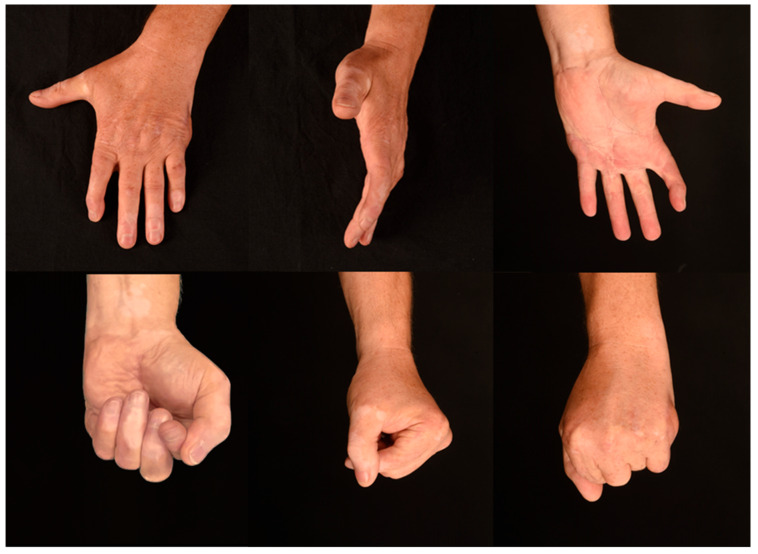
The outcome 11 months after transplantation of the vascularized lateral femoral condyle flap.

Eleven months post-surgery, minor restrictions of the index finger due to the arthrodesis and terminal flexion limitations of the little finger were observed. During the assessment of full-range of motion, the reconstructed fifth MCP-joint achieved 10 degrees in extension, 0 degrees in a neutral position, and 75 degrees in flexion. A table of fingertip-to-palm distances (FPD) can be found in Table 1. Figure 9 displays the images of the long-term follow-up 11 months after the reconstruction.

During the examination, the patient completed the Michigan Hand Outcome Questionnaire (MHQ) developed by Chung et al. [5]. Notably, there were particularly good results in the areas of activities in daily living and aesthetics, with no discernible difference compared to the dominant right hand. The overall hand function and satisfaction also showed outstanding results, but were slightly marked down due to the somewhat limited range of motion of the index finger, which the patient reported as bothersome in his profession as a blacksmith. The assessment of work performance was similar, but was marked down because the patient mentioned that tasks now take him longer. A comparison of the MHQ scores for the left hand versus the right hand can be found in Table 2. Overall, the patient is very satisfied and grateful that he can continue his life as before.

## 3. Discussion

Depending on their severity, hand injuries are one of the most common, involving a lengthy procedure consisting of multiple surgeries, physiotherapy, and countless follow-up visits. Therefore, well thought-out planning and exact execution of the upcoming reconstruction is of utmost importance to achieve a satisfactory outcome for the patient. Despite preoperative preparation and precise implementation, more than 50% of affected patients still suffer from functional limitations, which restrict their everyday life and work [1].

In recent years, a major achievement for reconstructive surgery is the production of replicas from a 3D printer, which enables a tangible representation of complex injuries for preoperative planning and simulation [3,4]. To date, most publications which focus on 3D-printed replicas for the preoperative planning are cases that report their proof of concept [6,7,8,9].

In addition to the acute treatment of a complex hand injury, our case reports the multistage procedure, including planning and execution, of MCP joint reconstruction by using a vascularized femoral condyle flap, which is considered ideal for small bone defects [10,11]. Based on the preoperative planning by using an individually produced 3D model, we opted to utilize the graft from the lateral femoral condyle instead of the initially intended one from the medial femoral condyle, as it was more suitable for the reconstruction of the metacarpal head due to the curvature of the affected bone. Both the medial and the lateral vascularized femoral condyle flap can be harvested as corticoperiosteal, corticocancellous, or osteochondral flaps, with or without an additional skin paddle. Further benefits are the variable size of these flaps, while maintaining a uniform vascular architecture, which enable harvesting of thin, small, convex grafts, easily adaptable for various reconstructive purposes. Therefore, these flaps provide various configurations of vascularized bone with nominal donor site morbidity [10,12]. Fundamentally, vascularized flaps from the femoral condyles are primarily utilized in the reconstruction of carpal disorders, in particular scaphoid and lunate, yielding proven outcomes [11,13,14,15,16]. Additionally, case reports of successful reconstruction of the ulna [17,18], radius [17,19], clavicle [20,21,22], and talus [23,24] using femoral condyle flaps can be found in the current literature. A few reports can also be found addressing the reconstruction of metacarpal bones [25,26,27,28]. In our case we demonstrate the successful reconstruction of the fifth metacarpal bone head with recovery of the functionality of the metacarpophalangeal joint. Due to exact preoperative planning, we were able to perform a thorough surgery and thus optimize the postoperative outcome for the patient, who was able to return to his metalwork profession after only a few months. The individual treatment plan, which included intensive physical therapy, i.e., autonomous performance of proprioceptive range-of-motion exercises by the patient, was crucial in supporting his postoperative outcome. The results, reflecting the patient’s overall satisfaction and functional recovery, were measured by the MHQ, which is a validated Patient-Reported Outcome Measure (PROM) and serves as an excellent tool for evaluating hand conditions [5,29]. Overall, we report on the reconstruction of a metacarpophalangeal joint with successful functional recovery by using a vascularized flap from the lateral femoral condyle. Furthermore, this case report highlights the efficacy of integrating individualized 3D printing technology to plan complex reconstructions, creating promising opportunities for personalized and optimized interventions.

## Figures and Tables

**Table 1:** The FPD is a clinical assessment used to evaluate hand function and limitations by measuring the distance from the fingertips to the palm as the patient tries to flex their fingers. This table displays the FPD outcomes for our patient’s impacted hand.

Fingertip-to-Palm-Distance	
Finger I	0 cm
Finger II	3.5 cm
Finger III	0 cm
Finger IV	0 cm
Finger V	1.5 cm

**Table 2:** The results of the MHQ in a side-by-side comparison. * indicates work performance and therefore applies to both hands.

Scales	Score	
	Left Hand	Right Hand
Overall hand function	95	100
Activities of daily living	100	100
Work performance	85 *	
Pain	30	0
Aesthetics	100	100
Satisfaction	95	100

## Data Availability

For data requests, please contact the corresponding author.
